# Mass Spectrometry Advances and Perspectives for the Characterization of Emerging Adoptive Cell Therapies

**DOI:** 10.3390/molecules25061396

**Published:** 2020-03-19

**Authors:** Camille Lombard-Banek, John E. Schiel

**Affiliations:** 1 National Institute of Standards and Technology, Gaithersburg, MD 20899, USA; john.schiel@nist.gov; 2 Institute for Bioscience and Biotechnology Research, Rockville, MD 20850, USA

**Keywords:** proteomics, CAR-T, biopharmaceutical, mass spectrometry

## Abstract

Adoptive cell therapy is an emerging anti-cancer modality, whereby the patient’s own immune cells are engineered to express T-cell receptor (TCR) or chimeric antigen receptor (CAR). CAR-T cell therapies have advanced the furthest, with recent approvals of two treatments by the Food and Drug Administration of Kymriah (trisagenlecleucel) and Yescarta (axicabtagene ciloleucel). Recent developments in proteomic analysis by mass spectrometry (MS) make this technology uniquely suited to enable the comprehensive identification and quantification of the relevant biochemical architecture of CAR-T cell therapies and fulfill current unmet needs for CAR-T product knowledge. These advances include improved sample preparation methods, enhanced separation technologies, and extension of MS-based proteomic to single cells. Innovative technologies such as proteomic analysis of raw material quality attributes (MQA) and final product quality attributes (PQA) may provide insights that could ultimately fuel development strategies and lead to broad implementation.

## 1. Introduction

Most of a cell’s phenotype and function are characterized by its proteome, the suite of all the proteins. The proteome is highly complex due to the vast protein dynamic range (6 to 10 orders of magnitude in concentration), the diversity of possible modifications (post-translational modifications, PTMs), and the variety of physicochemical properties of proteins. Therefore, most of the early proteome wide studies relied on the measurement of transcripts as a proxy to protein levels. Recent work, however, have highlighted the complex and dynamic relationship between RNA transcription and protein expression [1,2]. The ability to understand relationships between protein expressions and cellular function have been revolutionized by proteomic methods based on high-resolution separation sciences (e.g., liquid chromatography and/or capillary electrophoresis) coupled to high-resolution mass spectrometry (HRMS) instruments [3]. Fundamental proteomic research has unearthed a wealth of knowledge pertaining to disease pathways, identification of disease markers, and drug targets for the remediation of these studied diseases [4]. Proteomic studies of the human innate immune system have also become increasingly important to understanding the complex signaling patterns involved in responding to diseases [5]. For example, MS-based phosphoproteomics has identified signaling pathways and specific proteins that are involved in the maturation of primary T-cells [6,7] and the function of cytotoxic T-lymphocytes [8]. These same innate immune functions, such as adaptive T-cell response, are now being harnessed by the biopharmaceutical industry to treat various grievous illnesses as discussed thereafter. The next step in analytical evolution of proteomics is the adaptation and translation of proteomic-based measurements to provide detailed product knowledge on the development and refinement of related therapeutic products such as adoptive cell therapy.

Adoptive cell therapy (ACT) is a rapidly emerging anti-cancer approach, whereby the patient’s own immune cells—tumor-infiltrating lymphocytes or T-cells—are engineered to express T-cell receptors (TCRs) or chimeric antigen receptors (CARs) [9]. TCRs resemble naturally occurring T-cell receptors, whereas CARs are fully synthetic and their structure mixes the antigen recognition part of an antibody and the signaling domain of proximal T-cell receptors. CARs typically consist of a single chain variant (scFv, antigen recognition domain of an antibody) ectodomain, a transmembrane region that anchors the CAR to the cell membrane (passes the information on successful recognition to the intracellular endodomain), and a signaling endodomain (triggers immune response upon tumor recognition). The structure of TCRs is typically more sensitive to foreign antigens than CARs and can recognize intracellular antigenic compounds bound to major histocompatibility complexes (MHCs). CARs on the other hand, promote stronger immune response against the target malignancy. Comparison of these two ACT modalities has been reviewed elsewhere [10,11]. CAR-T cell therapy has advanced the furthest in clinical applications, with recent approvals by the Food and Drug Administration (FDA) of Kymriah™ (trisagenlecleucel) and Yescarta™ (axicabtagene ciloleucel) [12]. Kymriah received approval for the treatment of relapsed or refractory B-cell acute lymphoblastic leukemia and is pending application for use on relapsed of refractory B-cell lymphoma patients who are ineligible for autologous stem cell transplant. Yescarta is approved for the treatment of aggressive non-Hodgkin lymphoma. These promising new therapies represent a revolutionary advance in treating cancer in that their mechanisms of action are orthogonal to that of chemotherapeutic and monoclonal antibody (mAb)-based modalities. 

The expansion of adoptive cell therapy approaches will undoubtedly continue to grow with advances in manufacturing technology, reviewed recently in references [13,14]. A representative proposal, for example, is the use of healthy donor cell (allogeneic therapy) in place of the current practice to use the cancer patient’s own cells (autologous therapy) as the cellular starting material. Such a shift in the raw material sourcing may, among other potential advantages, ease patient burden and improve accessibility to a stock of starting cellular material. In order to fully understand the potential implications of such a shift and select the most appropriate production pathway for a given product requires process and product knowledge regarding attribute criticality as it relates to appropriate raw material quality assessment (MQA) and final product quality attributes (PQA). Full realization of the potential of both autologous and allogeneic CAR-T therapy would greatly benefit from adaptation and targeted innovation of analytical technologies to provide increased biomolecular understanding. Fluorescence activated cell sorting (FACS), for example, can provide invaluable information regarding transduction efficiency using highly specific anti-CAR antibodies to discriminate CAR-expressing cells. FACS is limited, however, because it relies on the production of highly specific antibodies and offers limited information on the global molecular state of the cell. Comprehensive identification and quantification of the relevant biochemical architecture represents a significant challenge and emerging high-resolution mass spectrometry approaches are poised as a major contributor to fulfilling CAR-T product knowledge needs. 

In this review, we are presenting the recent advances made in population and single-cell mass spectrometry-based proteomics that we deemed relevant for the characterization of CAR-T cell therapies. We provide an overview of the manufacturing process of CAR-T therapeutics, literature examples of proteomic-based measurements in CAR-T, and recent advances in proteomic technologies that are assured to extend its implementation. A focus will be given to advanced proteomic sample preparation strategies, micro and nanoscale separation science capabilities, the emerging field of single-cell proteomics, and a perspective on proteomic measurement controls that should be considered when developing proteomic methods. 

## 2. CAR T-Cell Therapy Manufacturing and State-of-The-Art Analytics 

### 2.1. Manufacturing of CAR-T

**Figure 1:**
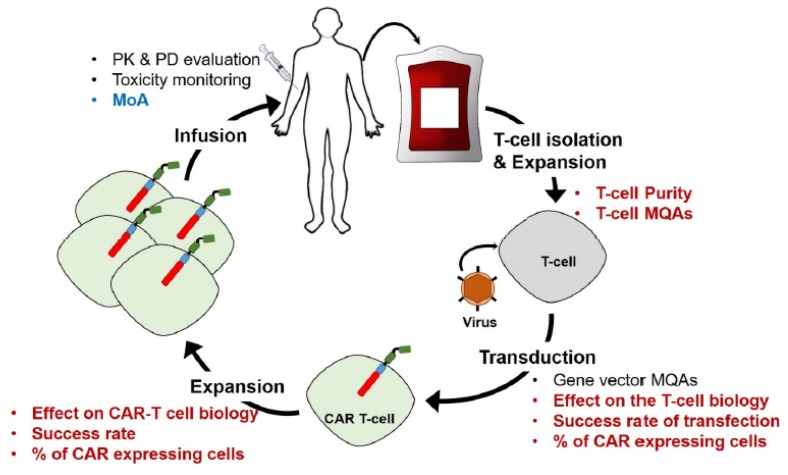
Schematic overview of the biomanufacturing of chimeric antigen receptor (CAR)-T cell therapies. The cycle starts with the isolation and expansion of T-cells from patient’s blood. The cells are then transduced to express the CAR by genetic engineering using a disarmed viral vector. Then the cells are expanded to multiply and differentiate into CAR-T cells targeting the select tumor antigen and are finally infused back into the patient. Steps of the manufacturing that would greatly beneficiate from MS-based proteomic characterization are highlighted in bold red font. The bold blue font indicates area where MS-based proteomics has previously been utilized.

CAR-T manufacturing is a highly complex process (Figure 1) and has been reviewed in detail in reference [14]. First, blood is collected from the patient themselves (autologous therapy) or from a healthy donor (allogeneic therapy). T-cells are separated from other blood components by leukapheresis. Leukapheresis is a medical procedure by which white blood cells (leukocytes) are harvested from the other blood components. Specific T-cells of interest are then purified by either size-based cell fractionation or specific antigen-carrying magnetic beads and further activated/expanded to increase their number. Isolated T-cells are genetically engineered to express the CAR on their surface using one of three major types of stable gene expression systems: γ-retroviral vector, lentiviral vectors, or the transposon/transposase system. The first two systems are the most widely used and rely on a disarmed virus to insert the CAR gene into the cells’ genome. The now engineered CAR-T cells go through an additional expansion step, whereby the CAR-producing cells are specifically multiplied using beads coated with the specific target tumor antigen or T-cell specific activation beads (e.g., Dynabead™ Human T-Activator CD3/CD28). After expansion, the cells are reintroduced into the patient where they can specifically target the tumor cells for which they have been engineered. 

CAR-T manufacturing would likely benefit from increased analytical characterization of the CAR-T proteome, and its potential dynamic evolution, during the manufacturing process steps as outlined in Figure 1 (red bullets). Successful starting cell populations may benefit from identification and control of material quality attributes (MQAs) (e.g., cell surface markers critical to transduction) and/or assurance of T-cell purity by demonstrating clearance of potentially contaminating cell populations (Figure 1). The transduction process is intended to bring about changes in protein expression, namely the CAR itself, which in theory can be monitored with quantitative proteomics. Proteomics would also offer the ability to simultaneously monitor for unintended changes in T-cell biology related to protein expression levels (e.g., epigenetic factors) and/or any potential deleterious effects from off-target protein expression. Similarly, each expansion step may result in proteome changes with increasing passage number that can be identified and understood. Finally, although certainly not least, final product quality attributes related to safety, potency, and efficacy may be identified via detailed proteomic studies (Figure 1). One could foresee in vitro screening bioassays for inter- and intra- cellular signaling cascades that might inform on mechanism of action (MoA), identify potential toxicity risks, and ultimately lead to improved rational product design. 

### 2.2. Overview of Mass Spectrometry-Based Proteomics

MS-based proteomic approaches are well-suited to fully understand the effect of cell engineering, cell expansion, and the mechanism of action of CAR-T cells because they can provide selective and sensitive proteome characterization without the use of antibodies for detection (Figure 1). Modern instrumentation has enabled the near to complete identification of yeast [15], *Xenopus laevis* embryos [16,17], mouse brain [18], and human proteomes [19,20]. Proteomic analysis may be conducted in an untargeted (i.e., discovery) fashion wherein putative identification and quantification of proteins are collected without a-priori knowledge of potential biological changes. Conversely, protein markers may be specifically monitored in a targeted proteomic analysis approach. Here we focus on untargeted approaches and how they can be used to help understand cellular biology. More information on targeted approaches can be found in other reviews [21,22]. 

**Figure 2:**
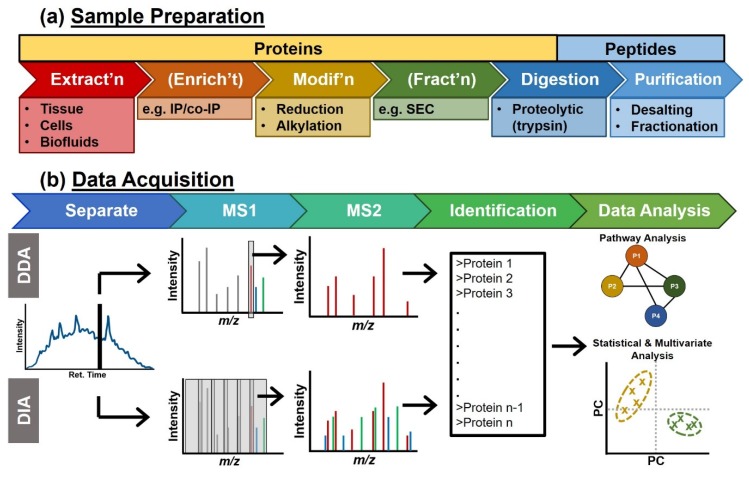
Standard MS-based bottom-up proteomic experiment. (**a**) First, biological samples are prepared via a bottom-up workflow, wherein the proteins are extracted from the cells and enzymatically digested into peptides. Key: IP, immuno-precipitation; SEC, size exclusion chromatography. (**b**) The resulting peptides are separated by reversed phase nanoliquid chromatography (nanoLC) and analyzed by mass spectrometry with a data-dependent acquisition method (DDA, top) or a data-independent acquisition method (DIA, bottom). A list of identified proteins is inferred from peptide sequencing using the observed MS2 fragmentation patterns. A variety of data analysis may be used. Statistical and multivariate analyses help in finding proteins that change between conditions or contribute to observed phenotypes. Over-representation and gene set enrichment analysis are used to place the obtained data in biological context. Key: Ret. Time, Retention Time; PC, principal component.

A typical sample preparation followed during bottom-up proteomics is presented in Figure 2a and additional information can be honed from references [23,24]. Briefly, proteins are extracted from cells in cultures, tissues, or biofluids following chemical and/or mechanical lysis of the cell membranes. The comprehensive suite of proteins can be extracted via organic solvent-assisted precipitation or filtration [3,24]. Alternatively, subsets of proteins (e.g., those that contain a specific post-translational modification or interact with a specific binding partner) can be enriched with various purification schemes (e.g., immunoprecipitation), as reviewed in [25,26,27]. The protein fraction is then prepared for digestion by reduction of disulfide bonds and alkylation of cysteine residues to prevent disulfide bridge shuffling/re-formation in solution. Proteins may be further fractionated (e.g., SDS-PAGE, size exclusion chromatography, etc.) and are enzymatically digested into peptides using a proteolytic enzyme that selectively cleaves at specific residues within a protein sequence (e.g., Trypsin cleaves after Arg and Lys) to prepare peptides of ideal size for mass spectrometry analysis. Samples prepared from whole cell or tissue population are typically complex, comprised of thousands of proteins and hundreds of thousands of peptides. Peptides are therefore often fractionated into multiple samples based on hydrophobicity and/or peptides containing specific post-translational modifications (PTMs, e.g., phosphopeptides, glycopeptides, etc.). Finally, the processed sample is desalted and/or exchanged into a solution suitable for data acquisition using ultrahigh-performance liquid chromatography (LC) or capillary electrophoresis (CE) coupled to an electrospray ionization (ESI) tandem high-resolution mass spectrometer (HRMS/MS).

Peptide separation is most commonly achieved using reversed phase LC. Capillary electrophoresis, however, continues to emerge as a powerful complementary separation technique in bottom-up proteomic workflows [28,29]. In both cases, eluting peptides are ionized via ESI and mass analyzed by high-resolution hybrid MS, such as a quadrupole time-of-flight (qTOF) or quadrupole orbitrap (qOT). Peptides are sequenced using data-dependent acquisition (DDA) or data-independent acquisition (DIA) modes as depicted in Figure 2b. In DDA, an MS1 scan is acquired first to populate a transient list of parent ion *m/z* values. Individual peptide ion signals are sequentially selected based on their intensity for further fragmentation (Figure 2b, top). Most commonly in DIA, the MS1 scan is followed by a series of wide (e.g., 25 Th) overlapping MS2 fragmentation windows independent of the presence or absence of peptide ion signals in the MS1 scan. The MS2 spectra are therefore a chimera of all the selected peptides (Figure 2b, bottom) [30]. Other DIA approaches have been developed such as MS^E^ [31], multiplex (MSX) [32], or variable Sequential Window Acquisition of All Theoretical Fragment Ions (SWATH) [33], and have been reviewed elsewhere [30]. This increasingly utilized acquisition mode responds to the missing data (e.g., unselected parent ions) issue encountered with the stochastic parent ion selection in DDA [4,34]. 

Recent improvements in MS hardware have greatly improved the proteome coverage and quantification accuracy in bottom-up proteomics. The addition of gas phase separation with ion-mobility modalities greatly benefits proteomic analyses in both DDA and DIA modes. Ion mobility technologies, such as field-amplified ion mobility spectrometry (FAIMS) [35], structures for lossless ion manipulation (SLIM) [36], traveling wave ion mobility spectrometry (TWIMS) [37], or trapped ion mobility spectrometry (TIMS) [38] add another separation dimension, which increases the peak capacity and provides additional information about the precursor peptide ions [39,40].

Protein identifications are achieved using bioinformatics software tailored to suite the acquisition mode. MS2 fragmentation data arising from each of the data acquisition modes corresponds to peptide-specific amino acid sequences of parent ions. In DDA, raw MS2 spectra are matched using a search engine to in silico produced spectra, derived from protein sequence of readily available databases (e.g., Uniprot [41]) and/or experimentally determined RNA expression [17,42]. Accepted software packages, such as ProteinPilot (ABScieX), Proteome Discoverer (Thermo Scientific), or MaxQuant [43] execute well-established search engines (SEQUEST [44], Mascot [45], Andromeda [46], or Paragon [47]) through user friendly interfaces. More details on DDA protein identifications strategies are available elsewhere [3]. In DIA, raw MS2 spectra must be deconvoluted to infer peptide and thus protein identities through two main approaches: peptide centric or spectrum centric scoring [30]. Peptide centric is currently the most widely developed approach. First, a spectral library is built from DDA data, which encompasses peptide query parameters (PQPs), including peptide retention times, peptide fragmentation pattern, and fragment relative intensities. Specialty software such as Skyline [48], OpenSWATH [49], or PeakView (SCIEX) assist in the creation of the PQPs from libraries and the subsequent extraction of peptide/protein identifications from DIA data. The rise of machine learning platforms to create spectral libraries in silico, promises to greatly alleviate some of the current limitations in creating spectral libraries and peptide query parameters for DIA data processing [50,51]. More recently, approaches to directly extract information from DIA data have been developed. For example, DIAUmpire deconvolute DIA data and convert the data to a pseudo DDA format, enabling protein identification using DDA processing platforms [52].

In untargeted bottom-up proteomics, hundreds to thousands of proteins are quantified in an experiment across different conditions (e.g., engineered vs. control T-cells). In DDA, relative abundance of each protein can be calculated from label-free quantitative information [53,54] or samples to be compared can be differentially labeled (e.g., isotope labels) for increased throughput, as reviewed recently elsewhere [34,55]. Label free quantification (LFQ) of proteins is based on spectral counting or ion signal abundance [56]. More recent approaches rely on advanced algorithm to infer LFQ values, such as the one implemented into MaxQuant: MaxLFQ [53]. LFQ combined with reference protein spike-ins [57] or protein ruler [58] enable semi-absolute quantification of proteins in samples. Alternatively, proteins or peptides are differentially labeled (or barcoded) across different conditions to be compared. Using stable isotopes labeling by amino acids in cell culture (SILAC), proteins metabolically incorporates isotopically labeled amino acids [59]. On the other hand, designer mass tags are used to differently barcode peptides obtained from enzymatic digestion. Two designs are commercially available: tandem mass tags (TMTs) [60] and isobaric tags for relative and absolute quantification (iTRAQ) [61], and other designs, such as di-leucine (DiLeu) [62] or the sulfoxide-based isobaric reagents [63] are synthesized in house. In DIA, proteins are quantified via a label-free approach whereby the area-under-the-curve of the elution profile of select fragment ions is measured [30]. Recently, DIA quantitative strategy has been multiplexed using SILAC [64]. With the use of alternative quantification methods using designer mass tags, such as the complementary ion reporter approach [65], similar strategies as used in DDA to multiplex sample analysis could be applied [34]. This approach would, however, necessitate drastic improvements in the computation of the generated data. 

Modern proteomic approaches identify and quantify thousands of proteins from cell culture and tissue samples; however, further interpretation of these data is required to shed light on their biological relevance and significance. Quantitative data are used to compare protein quantities across different conditions. Relative abundances are used to perform statistical and/or multivariate analysis to tease apart proteins that differentiate conditions. For example, student’s t-test is commonly performed to identify proteins that are differentially expressed with statistical significance (P-value <0.05) between a control and a perturbation test. Multivariate analyses (e.g., principal component analysis) derive a subset of proteins from the entirety of the proteomic data, which expression patterns contribute in differentiating experimental conditions or phenotypes. Results from statistical tests must be interpreted to enable biological conclusions. Tools enabling over-representation analysis such as gene ontology (GO) annotation, gene set enrichment analysis, or protein network inference help with relating observed protein changes to biological mechanisms. Multiple online platforms facilitate these analyses. For example, PantherDB [66] and David [67] enable GO annotation based on protein/gene names, KEGG [68] or Ingenuity Pathway Analysis (Agilent) infer on gene set enrichment, and STRING [69] and recently BioPlex [70] can help build protein interaction networks. 

### 2.3. Mass Spectrometry to Decipher the Mechanism of Action of CAR-T Therapies

Proteomic analyses of CAR-T have begun to surface in the literature, with seminal papers dedicated to the interrogation of the MoA of CAR activation (Figure 1, blue bullet). Coupling bottom-up proteomics with a phosphopeptide enrichment strategy, Salter and co-workers identified various pathways involved in kinase-mediated CAR-T cell signaling [71]. The authors compared the signaling events generated by two different CD19 targeting CAR designs that differ by their co-stimulatory domains, CD28/CD3ζ and 4-1BB/CD3ζ, currently in clinical trials as B-cell malignancy treatment. Salter et al. found that the signaling pathways represented by the identified phosphoproteins did not differ, but that the timing and strength of the signal varied. CD28 CAR led to a faster response, a more potent activity, but less persistent activation than the 4-1BB CAR design [71]. Such information, coupled with clinical data, could be critical to fuel future product design and to understand the interplay between potency and pharmacokinetics. 

In a more recent study, Romello and co-workers combined transcriptomics, immunoprecipitation (IP) MS to identify pathways that are differently regulated upon expression of second or third generation anti-prostate stem cell antigen (PSCA) CARs and then performed untargeted phosphoproteomics to identified signaling pathway differences upon CAR activation [72]. Second and third CAR designs essentially differ by the structure/composition of their intracellular signaling endodomains. The authors identified changes in protein expression profiles of CAR engineered T-cell compared to a GFP expression control, that demonstrated “tonic signaling” from CAR-T cells. “Tonic signaling” is a signaling cascade delivered in the absence of the target antigen and has been associated with poor CAR-T persistence in-vivo. Romello et al. showed that “tonic signaling” was more prevalent with the second-generation CAR design. Using phosphoproteomic analysis after subjecting the CAR-T cells to antigen recognition, the authors found that the second-generation design led to stronger signaling (types of proteins and relative intensities) compared to the third generation CAR. These results are of tremendous benefit for the design of new, safer, and more potent CAR molecules. This work represents an example where LC-MS based proteomics uncovered important changes upon CAR expression and identified co-stimulatory factors [72]. 

These two recent studies pave the way to improve understanding of the CAR activation mechanism and the MoA of CAR-T therapies. It is the authors’ contention that proteomic strategies applied to CAR-T will increase in frequency as well as scope in the coming years as discussed in Section 2.1. It comes as no surprise that literature reports of proteomic data on adoptive cell therapy are limited to a few pioneering papers when one considers the relative novelty of this treatment modality and the fact that the associated information revealed may offer competitive advantages harbored as company-specific intellectual property. The innovative technologies and strategies being employed in other sectors of proteomics, however, provide the foundations that could tremendously benefit CAR-T applications whether public or private. Herein we have summarized a subset of innovations we consider pivotal to a broader deployment of CAR-T proteomics. 

## 3. Advances in Mass Spectrometry that Will Benefit CAR T-Cell Therapy Characterization

### 3.1. Advances in Sample Preparation

Sample preparation is a critical part of the bottom-up proteomic workflow (Figure 2a). The first step involves breaking the cells and efficiently extracting the protein content. Typically, strong detergents (e.g., sodium dodecyl sulfate) are used to lyse the cells and solubilize proteins. These chemicals are often, however, incompatible with MS analyses and require extensive cleaning steps (e.g., protein precipitation) prior to trypsin digestion, which lead to sample losses and biases in the type of protein detected [73,74]. Overall, limiting the number of transferring steps reduces adsorptive losses encountered during the bottom-up proteomic workflow. Recent advancements in proteomic sample preparation have been made to enable protein clean-up, digestion, followed by peptide desalting within a single device [73,75,76,77,78,79]. Table 1 summarizes some of these advances and compares their respective features, each of which will be discussed in more details.

Pioneering most of the advances in filter-based sample preparation, filter-aided sample preparation (FASP) typically uses 10 kDa molecular weight cut-off (MWCO) filters to retain proteins while enabling the removal of lower molecular weight detergents and salts (Table 1), but MWCO filters ranging from 1 to 100 kDa in size are available [73]. Enzymatic digestion is then performed on the filters themselves and the resulting peptides are released by centrifugation [73,75]. FASP showed great success in improving peptide identification rates compared to the traditional in-solution approach and demonstrated a higher number of membrane proteins identified than previously obtained via the classical in-solution method [73]. This approach has, however, multiple drawbacks: FASP is labor intensive and requires substantial amounts of material. Approaches accommodating smaller amounts of protein material are needed to promote better proteomic characterization of limited biological samples, such as tissue biopsies or rare cell samples. 

**Figure 3:**
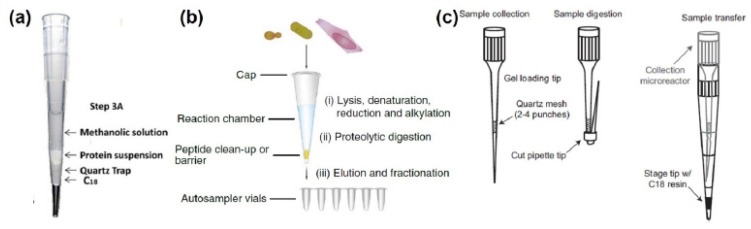
Advanced proteomic sample preparation for bulk MS-base proteomic analyses. (**a**) Representation of the suspension trapping (STrap) tip device. Adapted from reference [78] (**b**) In-Stage tip (iST) design for lysis and protein processing. Adapted from reference [77] (**c**) Combined S-Trap and iST design for streamlined proteomic sample processing for the analysis of fluorescence activated cell (FAC)-sorted cells. Adapted from reference [80].

Protein lysis and peptide clean-up are performed in separate receptacles in FASP, creating a potential source for adsorptive losses. Methods enabling the majority of the sample preparation to be performed in the device are especially well suited for sample minimization. For example, suspension trapping (S-trap) sample preparation device enables the clean-up, digestion, and fractionation of peptides within a single device (Figure 3a) [78]. The S-trap device is a design for centrifugation-based workflow and is composed of a quartz filter to trap intact proteins followed by a C18 (reversed-phase) monolith plug onto which the peptides are retained and sample desalting and/or fractionation is performed. The S-trap-based sample processing is simplified and faster compared to the FASP protocol due to its integrated design. Acidified cell lysate is first added to the column in the presence of a trapping buffer. A protein suspension is formed and is trapped in the quartz trap after centrifugation (see Figure 3a). The sample is then washed with the trapping buffer to remove detergents and chaotropic agents that could interfere with enzymatic digestion and mass spectrometric analysis. Typical buffer for enzymatic digestion, such as an ammonium bicarbonate solution, and the enzyme of choice are added onto the device to digest the trapped proteins. The resulting peptides are washed with ammonium bicarbonate/trifluoroacetic acid solution and captured onto the C18 plug. Peptides are released using a high organic (acetonitrile or methanol) solution thereby allowing desalting and/or fractionation prior to MS analysis. Sample as low as few 100 s ng of protein material can be prepared and the approach is compatible with a wide range of detergent [82,83].

Another example, with in Stage-Tip (iST) sample preparation, cells can be lysed in the device directly (Figure 3b) [77]. The iST device is simply a pipette tip with a C18 disc insert that serves as barrier and peptide clean up device. Thanks to the tip design, the preparation of multiple samples can be parallelized with the use of multichannel pipettes or even automated using robotic devices. Indeed, the entire processing can be done in a 96-well device, tremendously improving sample preparation throughput. One caveat of iST is the requirement for MS friendly detergent, as the C18 plug is unable to remove larger/ionic detergents like SDS. Moreover, the sample is heated at high temperature prior to digestion (>60 °C), preventing the use of chaotropic agent such as urea. In a study comparing, iST to FASP, iST led to a better coverage of membrane and nuclear proteins compared to FASP [77]. 

Most recently, filter-based proteomic preparation was revised to enable the processing of FAC sorted immune cells (Figure 3c). The device resembles a decoupled version of the suspension trapping (Strap) design in the format of a tip like the iST design (Figure 3c, left) [80]. The cells are collected during sorting directly in the quartz tip and retained by the quartz mesh. The quartz tip is then bent to avoid losses during digestion and the fold is maintained by a ring cut out from a pipette tip. The cells are lysed, and the extracted protein digested (Figure 3c, middle). The preparation tip is then inserted into a second tip containing a C18 plug for peptide clean up (StageTip). In their study, Myers and co-workers also performed labeling of the samples with designer mass tags inside the StageTips, which enabled relative quantification of up to ten samples [80]. This approach allowed for the preparation for quantitative analysis of samples containing less than 2 μg of protein content, representing an ~5 to 100-fold decrease in sample requirement compared to traditional MS approaches (Table 1).

**Figure 4:**
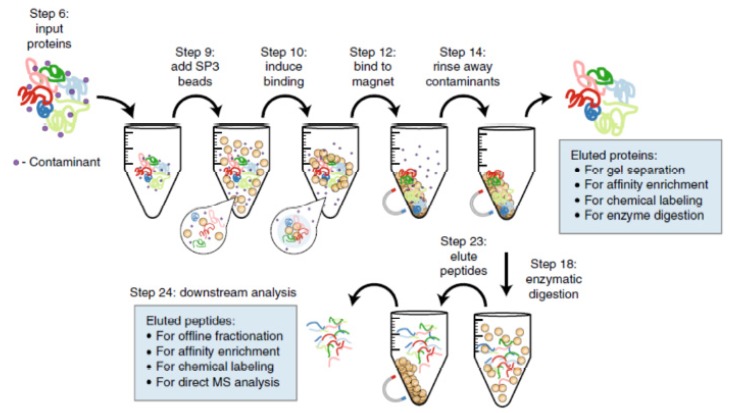
Single pot solid phase-enhanced sample preparation (SP3) for proteomic analysis. Reproduced from reference [81].

Lastly, building on the classical sample preparation approach taking place in a microcentrifuge tube, single-pot, solid-phase-enhanced sample preparation (SP3) uses magnetic beads derivatized with hydrophilic functional groups (Figure 4) [76,81]. This approach enables protein enrichment, detergent removal, and digestion in solution phase by adding the beads directly in the cell lysate formed in a microcentrifuge tube (Figure 4). All the sample processing steps are therefore performed in a single tube and the cost of the assay is lower than that of the approaches described previously. This approach is compatible with a wide range of detergent and chaotropic agents [81]. Moreover, it is compatible with a wide range of sample inputs, from very low to high, by solely changing the size of the microcentrifuge tube used and the amount of magnetic beads added [81,84,85]. 

Each of these approaches are highly complementary and result in a more streamlined bottom-up proteomic workflow compatible with low protein quantities (Table 1). Although these approaches have not yet been evaluated for systems relevant to drug development, multiple studies are available, which compare and contrast the different approaches for different cell lines [74,85,86]. For instance, Sielaff and co-workers compared FASP, SP3, and iST for the proteomic analysis of HeLa cells and FAC-sorted macrophages [85]. The authors found that SP3 and iST led to similar protein identification numbers (ID rates), independent of the amount of starting material. When comparing the performances of these three approaches for limited amount of starting protein material (~2.5 × 10^5^ cells or ~1 µg of protein material); however, Sielaff et al. demonstrated that SP3 outperformed FASP and iST. In particular, FASP performed particularly poorly for sample <2 µg, probably due to the less streamlined workflow that can increase losses [85]. In a separate study, two filter-based approaches, FASP and S-trap, were compared with the traditional in-solution protocol [86]. The two filter-based digestion approaches performed similarly and were overall better than in solution digestion. Indeed, FASP and S-trap led to a lower number of peptides with missed cleavages and produced greater ID rates. In particular, S-trap with urea-based lysis buffer led to the highest number of proteins identified. Moreover, Strap based approaches enabled the identification of proteins that covered a greater panel of cellular components. However, some proteins were found to be differently enriched with the different preparation methods [86], suggesting that careful evaluation of the protein extraction and digestion workflow must be considered for specific questions and applications. 

The modernized sample preparation approaches in Table 1 promise to further the understanding of several aspects of CAR-T cell therapies. First, each of these approaches enhance the coverage of the membrane proteome identified. The CAR is anchored in the membrane of the engineered T-cell and that part of the signaling cascade unraveled by antigenic activation of the CAR involves membrane bound proteins. Improving the extraction of this class of proteins is therefore critical to improve the understanding of 1) the mechanism of action of CAR-T cells and 2) allow the identification/characterization of PQAs. Second, these approaches enable the handling of limited protein amounts, a critical feature when analyzing precious samples such as patient derived cells. Finally, the overall improvement in the number of proteins identified promises to expand the identification and understanding of MQAs (T-cells) and PQAs (CAR-T). Such information will help design better CARs to enhance efficiency and safety of this therapeutic modality. 

### 3.2. Advances in Peptide Separation

Bottom-up proteomic sample preparation creates thousands to millions of different peptide species embedded in complex matrices to be thereafter analyzed by mass spectrometry. To enable the successful analysis of such a large number of peptides, separation prior to ionization and MS analysis and detection is necessary. Modern proteomic analyses utilized liquid-based separation approaches to resolve peptide species. Moreover, for the analysis of clinically relevant samples, separation techniques amenable to low sample input are needed. Two figures of merit of separation techniques that are critical to improve the analytical workflow sensitivity are scale and resolution. Nanoscale separations are categorized by small inner diameter columns/capillaries, a nL/min flowrate, and concurrently small electrospray nebulizer. Flowrate dictates how much dilution the sample encounters during separation, and thereby lower flowrates typically lead to higher sensitivity. In addition, electrospray at nL/min flow rates results in significantly smaller droplets, which are more efficiently desolvated/ionized; thereby, significantly enhancing limits of detection and sensitivity versus µL/min systems. Resolution is a measure of how well species get separated from each other. Separation techniques with better resolution decrease the number of coeluting peptides, which in turn reduces ion suppression and improves the use of the MS duty cycle. New technologies for the liquid-based separation of peptides have tremendously improved the analytical sensitivity of bottom-up proteomic approaches. 

**Figure 5:**
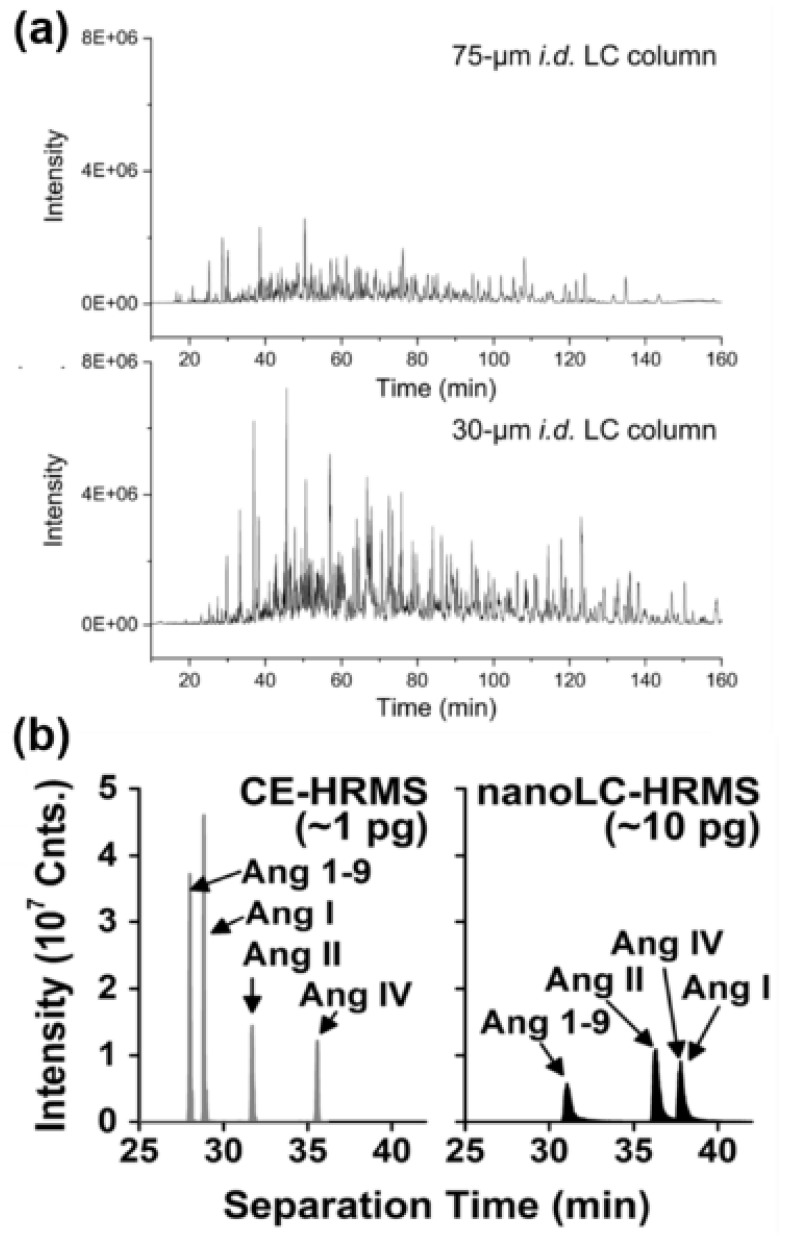
Advanced separation modalities for the sensitive analysis of peptides and protein from low-sample input. (**a**) Reduction of the capillary diameter for reversed-phase chromatography enhances the peptide signal in shot-gun proteomic analysis. Figure adapted with permission from reference [87]. (**b**) Capillary electrophoresis (CE) enables ultra-sensitive analysis of peptides. Adapted with permission from reference [94].

Reversed-phase nanoliquid chromatography (nanoLC) is the gold standard separation in MS-based bottom-up proteomic analysis. It is easily coupled to MS via electrospray ionization (ESI) and multiple commercial options are available. However, until recently, nanoLC approaches were only modestly compatible with the analysis of limited sample input. Recent development in reverse-phase nanoLC columns have tremendously improved the use of nanoLC for mass-limited protein sample analysis. For instance, the careful reduction of the LC support particle size and capillary column diameter led to notable improvement in peak height and therefore improved signal-to-noise ratio (Figure 5a) [87]. The reduction of the capillary inner diameter from 75 µm down to 30 µm resulted in an ~2-fold improvement in peptide signal (Figure 5a) [87]. This development coupled with further advances in sample preparation (discussed later) enabled the proteomic analysis of ten to hundreds of mammalian cells (HeLa) and tissue laser microdissections encompassing ~10 to 50 cells to unprecedented depth [88,89]. The development of alternate reversed phase column formats offers great outlook for the analysis of amount-limited protein samples. For example, custom porous layer open tubular (PLOT) columns enabled the detection of bovine serum albumin (BSA) peptides at 10 zmol level [90]. PLOT columns are borrowed technology from gas chromatography. They are made of an open capillary tubing and the walls are coated with a material capable of interacting with the analyte of interest. In the work of Li and coworkers, the 4 m long and 10 µm diameter column walls were comprised of poly(styrene-divinylbenzene). This technology enabled the identification of ~1,300 protein groups from ~50 mammalian cells, which represented a 4 to 5-fold improvement in sensitivity compared to state-of-the-art nanoLC-MS approaches [90,91,92]. Most recently, micro pillar array (µPAC) nanoLC columns have been developed. µPAC columns are manufactured by a lithographic etching process of posts on a silicon chip that can be then be derivatized with C18 groups for reversed phase. This LC on a chip technology creates a perfectly ordered separation bed that displays tremendous separation efficiency, with plate number approaching that of capillary electrophoresis [93].

Capillary electrophoresis (CE) has emerged as an alternative approach for the ultra-sensitive analysis of proteomic samples. CE displays exquisite peak capacity and high plate numbers, which therefore tremendously boosts the sensitivity. Using CE separation prior to MS detection, sensitivity in the ~100 s zmol have been demonstrated [94,95]. Most recently, using BSA coated vials, Zhenbin et al. showed lower limit of quantification as low as 1 zmol for a standard angiotensin peptide [96]. Figure 5b illustrates the sensitivity gained by CE compared to nanoLC for a set of model angiotensin peptides. While measuring 10-times less material, the peptide signals intensities from CE measurements are twice as high as one obtained with nanoLC [94]. Moreover, all four peptides contained in the mixture were resolved using CE, while angiotensin (Ang) I and IV co-eluted by nanoLC (Figure 5b). Multiple reports have demonstrated that CE coupled to MS is highly complementary to conventional nanoLC for bottom-up proteomic analysis of limited samples [94,97,98,99]. Coupling of CE with electrospray ionization is not trivial, however multiple designs have been developed and are reviewed in references [29,100]. Furthermore, CE separation is typically more compact in time than LC. Peptides migrate through the capillaries within a few minutes, often challenging the MS duty cycle [95,101]. To remediate this challenge, separation at lower voltage is possible, albeit at the expense of the high-separation power offered by CE. Recently however, Chen et al. enabled the separation of peptides over ~2 h window by suppressing the electroosmotic flow and revising the sample reconstitution solution [102].

The examples described here clearly show the inherent advantage of nanoflow separation science toward depth of proteome characterization. The ability to analyze increasingly small sample sizes is imperative for CAR-T therapy when one considers that each cell analyzed is a cell that ultimately does not reach the patient. The use of nanoLC and CE allows minimal sample consumption while simultaneously affording the highest proteome coverage. CE and nanoLC, along with the increasing sensitivity of modern mass spectrometers, is pushing the boundaries of sample minimization even further–toward single cell analysis. 

### 3.3. A New Venue for MS-Based Proteomics: Single-cell Analysis

Measuring the proteome with single cell resolution is a substantial analytical challenge. Proteins, unlike nucleic acids, do not benefit from amplification reactions. Therefore, the sensitivity of measurement solely relies on the effectiveness of the sample preparation workflow and lower limit of detection/quantification of the analytical platform. Currently, protein measurements in single cells are performed via targeted antibody-based assays such as single-cell western-blots [103] and mass cytometry (CyTOF) [104]. Although powerful, these approaches are limited to the measurement of small numbers of proteins (max. 100 proteins), require previous knowledge about the protein to target, and their validity is dependent on the quality of the antibodies used for the assay [105]. Conversely, bottom-up proteomics by MS can measure thousands of proteins without a priori knowledge, potentially revealing mechanism of diseases [3]. In the past five years, great strides have been made to extend the application of MS-based proteomic to the measurement of single cells. This application was rendered possible by advances in sample preparation, enhanced nanoscale separation modalities discussed in Section 3.2, and improvement in MS instrumentation [106,107,108]. Single cell handling and sample preparation for proteomic analysis is perhaps the most challenging step of the workflow and requires more advanced technology than described in Section 3.1 to accommodate for the much-reduced protein material afforded by single cells. Proteins tend to absorb readily onto the surface of containers used for standard preparation. This phenomenon is exacerbated as the protein concentration decreases, generating considerable losses when handling protein amount limited samples. Despite this phenomenon, using approaches downscaled from bulk analysis, ~100 s to thousands of proteins have been identified from larger cells, such as dissected *X. laevis* embryonic cells [99,101,109] and human oocytes [84]. 

**Figure 6:**
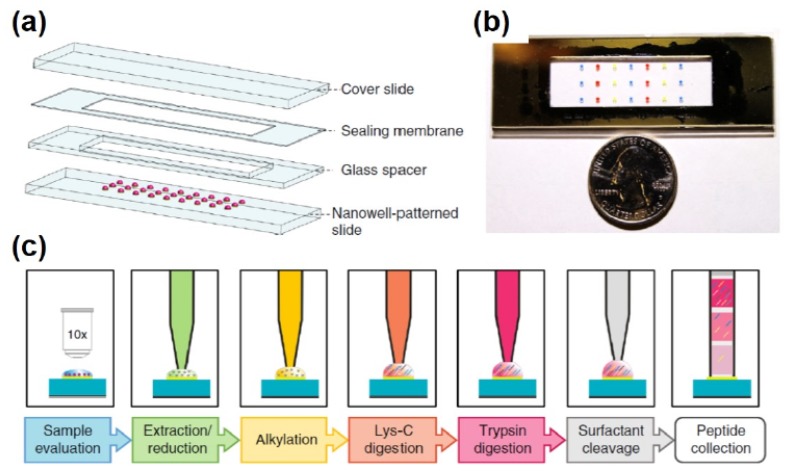
Nanodroplet processing in one-pot for trace proteomic samples (nanoPOTs) enables the label free proteomic analysis of single cells. Schematic (**a**) and picture (**b**) representation of the nanoPOTs glass chip. (**c**) Sample processing workflow using the nanoPOTs approach. Reproduced with permission from reference [88].

Over the past ~5 years, great strides have been made to miniaturize and streamline the preparation of single cells further [106,110]. The common idea in all the sample preparation approaches that were developed to enable single cell proteomic analysis is 1) downscaling of the reagent volumes and 2) limiting the sample handling by performing the preparation in a single container. Using this concept, Lombard and co-workers extended MS to probe the proteome of single cells, with subcellular resolution for embryonic cells from *X. laevis* and zebrafish embryos [111] and mouse neurons [112]. Using nanoliter-scale oil air droplet (OAD) chip, single HeLa cells and mouse oocytes were prepared for bottom-up analysis on an orbitrap instrument leading to the identification of ~40 to ~250 proteins on average, respectively [113]. Zhu et al. further downscaled and partially automated the process for proteomic sample handling with the development of the nanodroplet processing in one-pot for trace proteomic samples (nanoPOTs) (Figure 6) [88,114]. The NanoPOTs device consists of microfabricated glass chips with photolithographed hydrophilic pedestals (Figure 6ab). The cells and reagents were delivered using a robotic platform with submicron positioning capabilities and able to handle picoliter volumes (Figure 6c). The entire procedure was performed within a ~200 nL droplet. The sample is then collected in a fused silica capillary, which can be stored until analysis after sealing the ends. This setup minimized sample losses due to transfer of the sample into autosampler vials. The capillary containing the sample is easily coupled to an SPE-clean up column and LC-MS analysis using standard microfluid fittings [88]. NanoPOTs, combined with advanced reversed-phase chromatography column and state-of-the-art MS instrumentation, has advanced the number of protein identified from a single HeLa cell to ~650 [114]. The approach was then applied to differentiate primary human lung epithelial and mesenchymal cells based on their proteomes [114]. Although powerful, using these approaches, each cell measurement takes 2 to 6 h, hindering throughput and thereby the measurement of a large number of cells necessary for meaningful statistical analysis. 

**Figure 7:**
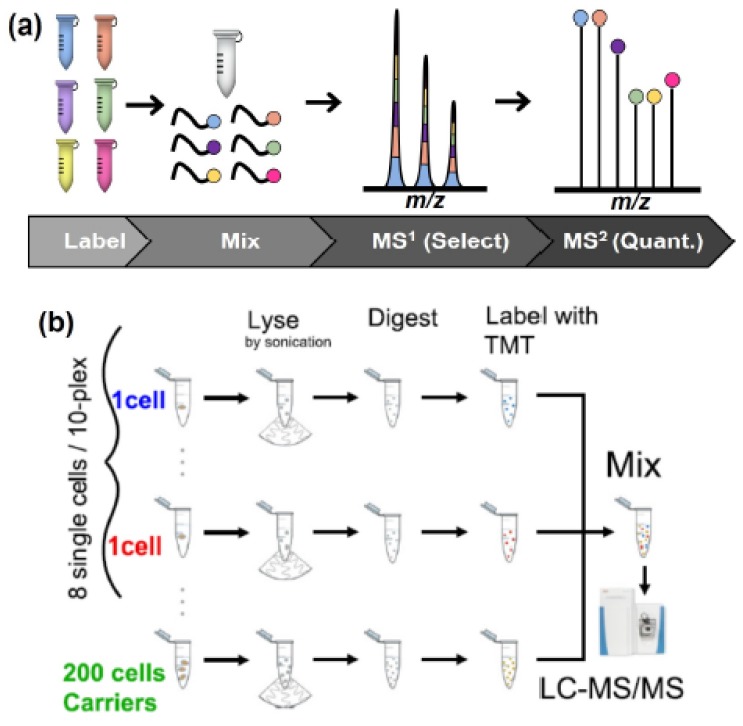
Multiplex analysis of mammalian cell for increased throughput and sensitivity. (**a**) Overview of the principle underlying multiplexed quantification with designer mass tags. (**b**) Single-cell proteomics by mass spectrometry (ScoPE-MS) enables the analysis of eight cells together. Reproduced from reference [119].

Paralleling the barcoding approach used in single-cell RNA sequencing (scRNA-seq) technologies [115,116], designer-mass-tags such as tandem mass tags (TMTs) [60], or isobaric tags for relative and absolute quantification (iTRAQ) [61] enable the measurements of multiple samples at the same time. These approaches have recently been reviewed in detail in reference [55]. Figure 7a describes the principle of relative quantification using designer mass tags wherein individual samples are tagged with different labels and then mixed prior to analysis. During MS analysis, the *m/z* signatures of the peptides from each sample are indistinguishable at the MS1 level. Therefore, the MS1 signal intensity of a given peptide is the sum of the intensity of the same peptide from all the samples. The quantitative information is acquired from the release of a signature fragment at the MS2 [60,65] or MS3 level [117,118] and is related back to each sample/condition (Figure 7a). 

Building on the signal summation at the MS1 levels, which improves peptide detection and selection for fragmentation, Budnik et al. developed an approach whereby one of the channels is used to “amplify” the overall peptide ion signal from the sample (carrier channel) by comprising peptides signals originating from multiple cells (Figure 7b). Using single-cell proteomics by mass spectrometry (ScoPE-MS), up to eight single cells can be measured together. This approach could differentiate two different cells types in a proof-of-concept experiment. Moreover, ScoPE-MS enabled identification of cell-to-cell heterogeneity during the differentiation process of embryonic stem cells [119]. The same principle was then implemented with the nanoPOTS approach which improved the proteome coverage further [120]. In this work, the proteome amount to be used as carrier was revisited, and 50-times the amount estimated for a single cell was found to be the maximum to ensure quantitative accuracy. Using a model of three different cell types, nanoPOTS combined with multiplexing identified cell specific protein profiles, showcasing the validity of the approach. Using these multiplexed quantitative approaches, ~65 to 70 cells could be identified in a day, representing a throughput close to the first generation of single cell RNA-seq technologies using Fluidigm C1 platform [121]. Implementation of the increased multiplexing capabilities imparted by new reagents [122,123], the throughput will be improved further, potentially reaching the levels of state-of-the art scRNA-seq approaches. Moreover, further advances in mass spectrometry, both hardware and acquisition modes, promise to increase the coverage amenable to single cell proteomic technologies.

Single-cell proteomic analysis would greatly benefit CAR-T cell therapy research and qualification at all steps of the manufacturing process. Indeed, isolation, genetic engineering, and expansion steps results in an heterogenous population of cells at the end of the process. Multiple reports taking advantage of single cell RNA-seq or single cell cytokine assays have reported heterogenous T-cell populations and CAR-T populations based on molecular composition and behavior [124]. Application of the new advances in single cell proteomic analysis promise to provide more in-depth information on this cell-to-cell heterogeneity at the proteome level. Such knowledge may lead to a better understanding of PQA criticality, identification of desirable sub-cell populations, and ultimately design of CAR-T cell products that result in more desirable properties such as lower tonic signaling, longer in vivo persistence, higher avidity, etc. 

## 4. Implementation of Measurement Controls

LC-MS peptide mapping of mAb-based therapeutics has evolved from characterization towards quality control (QC), and has garnered widespread interest in the current state-of-the-art Multi-Attribute Method (MAM) [125]. Concurrently, the need for instrument control and system suitability has kept pace with translation of MAM analysis toward the QC environment [126]. To this point in this review we have predominantly been discussing proteomic measurements as a research tool to inform on process development and product characterization. One can envision, however, a futuristic goal of using proteomic analysis as a process and/or product development and/or control strategy. In all cases, stringent measurement standards must be in place and methods carefully validated and controlled to maximize utility of comparatively scarce cellular material. Consensus best practices for assay development (e.g., experimental design) and validation will be critical to the success of MS-based proteomics as a cell therapy characterization tool [4]. In this section we focus on existing best practices to evaluate instrumentation performances. 

**Figure 8:**
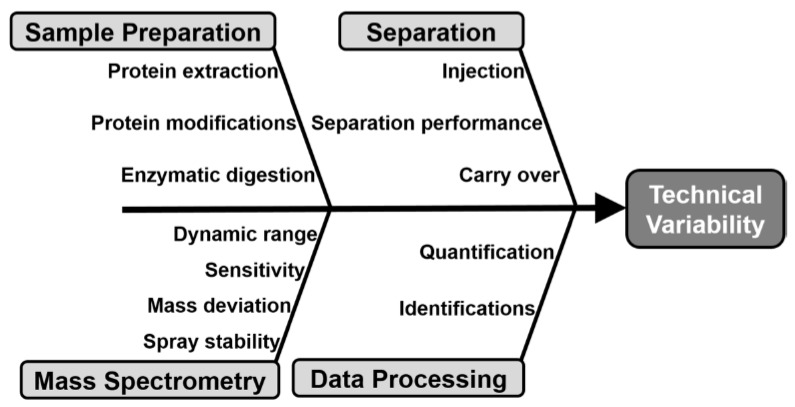
Fishbone diagram highlighting the principal sources of variability in a MS-based proteomic experiment that should be evaluated during system suitability assessment.

Indeed, during MS-based proteomic analysis, many factors may contribute to the technical variability as outlined in Figure 8 [127,128,129,130,131]. Many of the steps that contribute to measurement variability during bottom up proteomic analysis, related to the nanoLC-MS/MS or CE-MS/MS instrument performance (e.g., separation and mass spectrometer), can be controlled using quality control standards (QC) and principles borrowed from historical MAM and/or clinical proteomic analysis [128,131,132].

**Figure 9:**
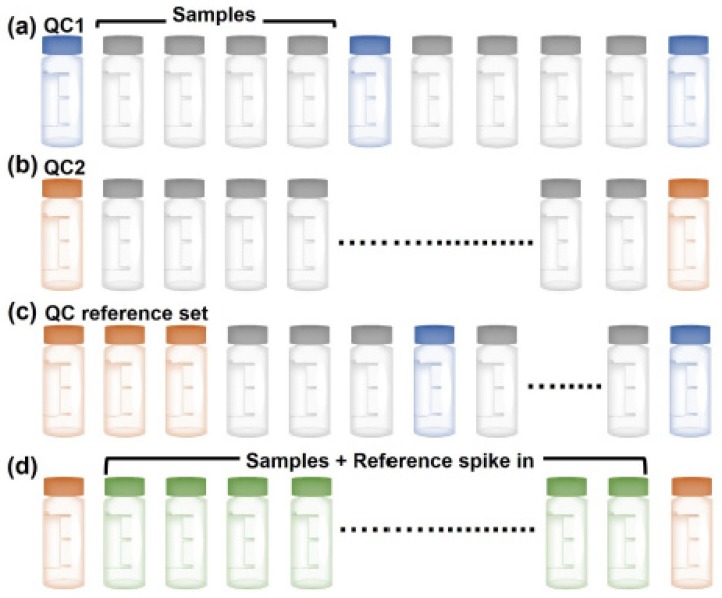
Standard experimental designs integrating quality control (QC) samples to evaluate the LC-MS/MS instrumentation for targeted or large-scale proteomic measurements. Simple QC samples including single peptide, peptide mix, or single protein digest (QC1) are in blue. More complex QCs comprising whole proteome digests (QC2) are in orange. Sample integrating a reference spike in are in green. The grey vials indicate the samples to be measured.

Approaches to evaluate LC-MS/MS instrument performance have been extensively reviewed [128,131]. The National Institute of Standards and Technology (NIST), in collaboration with the National Institute for Cancer (NCI), have identified 46 metrics, termed MSQC, for evaluation of the performance of LC-MS/MS systems, which can be divided in six categories: chromatography, dynamic sampling, ion source, MS1 signal, MS2 signal, and peptide identification [132]. To control for less than optimal instrument performance, quality control samples should be run. These samples range from a simple peptide mixture [133], to a single or mixture of protein digest [127,133,134], to a complex proteome digest [135,136]. When and at which frequency the QC standards should be measured is highly dependent of its nature [128,137]. Different experimental design that integrate QC runs that are most widely encountered are presented in Figure 9. Peptide mix or single protein digests are typically used to monitor nanoLC performances: peak shape and retention times. These types of QC are typically run more often, as they use short gradient times and are therefore not so detrimental to the measurement throughput (Figure 9a) [131,133]. Conversely, more complex pre-digested samples, such as full cell lysates, from yeast [135,136], HeLa [130], or *Pyrococcus furiosus* [138] help assess the MS performance. These complex samples monitor more parameters including mass accuracy, sensitivity, and dynamic range and may be less frequently interspersed (Figure 9b). Alternatively, a combination between analysis of a simple peptide mix QC sample and a more complex digests can be used to qualify the instrument both before the start of the measurement series and during the sequence (Figure 9c) [127]. In this instance, a high quality QC reference sample set, for example a complex digest, is measured prior to analysis of real sample to benchmark the instrument and shorter QCs (e.g., peptide mix) are run at set intervals during the sequence. Finally, spike in of a peptide standard mixture, such as synthetic, heavy labeled peptide, spanning a wide concentration range into every sample of interest can help assess the quality of each sample measurement (Figure 9d) [137]. 

Some informatics platforms have been developed to assess the quality of the QC sample injections and the instrument performance. For example, SprayQC and the development of ESI interface monitoring [139] allows for the visualization of the spray over time to address any issue linked to spray instability and improper Taylor cone formation that would indicate inefficient ion formation [140]. Statistical Process Control in Proteomics (SProCoP) is a tool, integrated into Skyline, an open software for the analysis of DIA and targeted experiments [127,141]. SProCop measured different metrics from QC runs and helps benchmark the instrument performances by comparing the data to empirically established thresholds [127]. Other approaches, such as QuaMeter [142] and NIST MSQC [132], help assess the system suitability of DDA data from QC runs. More recent tools, such as MSStatQC [143], help assess targeted measurements suitability. On the other hand, Proteomics QC (PTXQC) allows for the identification of outlier data sets from replicate runs, by evaluating both the number of identification and the peptide intensities across multiple runs extracted from MaxQuant generated data [144]. 

With the arising of multiplexing approaches for both bulk analysis and single cells using designer mass tags [60,61,122], the need to evaluate quantification accuracy and suitability of the MS to ensure the best results possible in terms of accuracy and sensitivity is ever increasing. To validate multiplexing experiments with tandem mass tags, both unit mass separated and isotopologue based, a triple knock out (TKO) yeast standard was developed [145,146]. This standard includes three different yeast strains, each deficient in a unique highly abundant protein. From this standard, an interference-free index (IFI) value is measured, where a high IFI is desired. From this measurement, separation, acquisition method, and state of the MS can be assessed. Moreover, an online open source platform exists to directly evaluate quantitative data obtained from TKO QC runs [147].

Each of these QC samples and informatics platforms will be pivotal in maintaining consistent proteomic results; however, an additional standard may also be necessary in order to have a true system suitability control that incorporates digestion efficiency. As highlighted earlier in this review, many sample preparation methods have been developed, both for population (Figure 4 and Figure 5) or single cells (Figure 6 and Figure 7), leading to widely different results depending on the applications [4,85,86]. Metrics to address appropriate sample preparation are for instance: number of protein identified/quantified; type of proteins identified; specific peptide detected for targeted quantification of protein of interest. To ensure smallest variation in measurements, best practices should be followed with the meticulous application of standard operating procedures, once such have been established. The proteomic workflow from sample preparation to data processing would most comprehensively be evaluated by a process- and product-representative in-house standard run periodically. The ideal in-house reference standard system suitability strategy for cell-based products is, however, undoubtedly complicated due to the nature of this product, and more so by the complexity of a proteomic measurement. Strategies from mAb-based biopharmaceutical lifecycle management can inform these decisions [148], but may require novel considerations for cell-based products.

## 5. Conclusions

Proteomic measurements have advanced our understanding of the underpinnings of cell biology to a degree thought unachievable a few decades ago. Meanwhile, the biopharmaceutical industry has developed a similarly innovative cancer therapy based on adoptive T-cell therapy. This fortuitous confluence of scientific achievements promises many synergistic advancements in the near future. Herein we have discussed the CAR-T development process alongside potential proteomic measurement implementation points. A series of advances in proteomic method sample preparation, separation science, and analysis down to the single cell level demonstrate the industrial readiness of proteomic methods in the development of CAR-T. A final discussion regarding potential control strategies, drawing from orthogonal proteomics research and monoclonal antibody-based therapies was also presented. 

Widespread implementation of proteomics-based measurements for CAR-T is attainable when considering advances in methodology and measurement control strategies. Molecular complexity is magnified significantly with cell therapies, yet adaptation and innovation of analytical methodologies such as proteomic measurements are poised to begin addressing critical questions regarding appropriate raw material quality assessment (MQA) as well as final product quality attributes (PQA). 

## Figures and Tables

**Table 1:** Comparison of modern sample preparation approaches for efficient bottom-up proteomics.

Sample Preparation	Digestion Location	Lysis on Device	Reagent Compatibility	Sample Input	Online Clean-up	Refs.
FASP	On filter	No	Wide variety of detergents and reagents	>20 μg	No	[73]
S-Trap	On filter	No	Wide variety of detergents and reagents	~500 ng–500 μg	Yes	[78]
iST	On filter	Yes	Cleavable detergent only	<1 μg–500 μg	Yes	[77]
Streamlined iST	On filter	Yes	Cleavable detergent only	<2 μg	Yes	[80]
SP3	In tube	Yes	Wide variety of detergents and reagents	100 ng–500 μg	No	[76,81]
